# Effectiveness of Experimental Whitening Toothpastes Containing Colorants on the Optical Properties of Enamel

**DOI:** 10.1155/2022/4576912

**Published:** 2022-03-31

**Authors:** Fabiano Vieira Vilhena, Carlos Frederico de Oliveira Graeff, Nádia da Rocha Svizero, Paulo Henrique Perlatti D'Alpino

**Affiliations:** ^1^Trials, Research and Development Inc., Bauru, SP, Brazil; ^2^DF-FC, UNESP–Universidade Estadual Paulista, POSMAT–Programa de Pós-Graduação em Ciência e Tecnologia de Materiais, Bauru, SP, Brazil; ^3^Hospital for Rehabilitation of Craniofacial Anomalies (HRAC-USP), Universidade de São Paulo, Bauru, SP, Brazil; ^4^Triplet Biotechnology Solutions, Bauru, SP, Brazil

## Abstract

**Objective:**

This in vitro study investigated the whitening potential of experimental toothpastes containing optical colorants in their formulations in comparison with commercial products. The chemical and physical characteristics of the toothpastes, the morphology, and elemental analysis of the enamel surface after treatment were also analyzed.

**Materials and Methods:**

One hundred twenty-five bovine incisor teeth were randomly divided into five groups according to the treatment: (i) experimental PHTALOX dental gel (PHT); (ii) experimental blue silica dental gel (SDG); (iii) Sensodyne Whitening Repair & Protect (WRP); (iv) Sensodyne True White (STW); (v) Snow White Toothpaste (SWS). The whiteness index differences (Δ*WI*_*D*_) and color alteration (CIEL_ab_-Δ*E*, CIEDE2000-Δ*E*_00_) were calculated after color change analysis using a spectrophotometer before and after the enamel treatment (*n* = 25). The surface and cross-sectional micromorphology were assessed using scanning electron microscopy. The elemental analyses were determined with energy-dispersive X-ray spectroscopy (EDS). The pH, particle size, zeta potential, and polydispersity index of toothpaste were evaluated. Data was statistically analyzed (ANOVA/Tukey, 5%).

**Results:**

Whitening toothpastes containing optical colorants were effective for whitening the enamel, as whiter teeth were observed following treatment (higher means of Δ*WI*_*D*_). In addition, when the parameters Δ*E*_ab_ and Δ*E*_00_ were evaluated, these toothpastes were graded as very good effectiveness (grade 4). Other toothpastes were graded as 3 (good effectiveness). PHT had a neutral pH and a larger mean particle size (412.8 nm). Elemental analysis demonstrated enamel with a silicon-enriched mineral layer on the enamel surface treated with SDG. The Ca/P ratio after enamel treatment varied from 1.74 (SWS) to 2.04 (SDG and WRP).

**Conclusions:**

Experimental whitening toothpastes containing optical colorants are effective at bleaching the enamel. The synergism among the different parameters analyzed seems to positively affect the color change after brushing with whitening toothpastes containing optical colorants.

## 1. Introduction

Although tooth whitening is usually characterized by the application of hydrogen peroxide in different formats, whitening toothpastes also contain specific abrasives and/or chemical agents in their formulation [1]. In this format, tooth whitening is dependent not only on the delivery of peroxides from the toothpaste to the tooth structures, but also on the removal and control of extrinsic stains [1, 2]. In spite of the limited contact periods during brushing [3], it is believed that the majority of bleaching toothpastes work by simultaneously whitening the teeth and/or by the removal and control of extrinsic stains [1]. In this manner, whitening toothpastes are reported to bleach the tooth color by about one or two shades [3].

Whitening using commercial toothpastes represents a simpler and cost-effective method [4] and was launched with the promise to being an alternative to home and/or dental whitening procedures with clinical results within 2 to 4 weeks [5]. In vivo studies demonstrated that the effectiveness of whitening toothpastes varies from 2 to 6 weeks, depending on the product characteristics [1, 6, 7]. Whitening toothpastes also offer therapeutic benefits (anticaries and antigingivitis) with added whitening activity from abrasives, adsorbent particles, peroxides, enzymes, or optical effect agents [8].

Modifications in the toothpaste formulations allowed the addition of a variety of novel active ingredients, which may alter the pH and abrasiveness of the products. Studies have demonstrated that abrasives are the most important components in toothpaste for stain removal [2, 9]. These abrasives help to remove extrinsic stains from the coronary surfaces, allowing clinically visible brighter alterations in tooth coloration [10]. For cosmetic reasons, manufacturers have used optical effects as a marketing strategy as many people prefer white teeth and a bright smile, which is invariably related to a higher quality of life [11].

As demonstrated in a previous in vitro study [12], the abrasives, which may react with the active ingredients, were found to affect the enamel surface, resulting in varying enamel surface mineral loss. Abrasives such as alumina, di-calcium phosphate di-hydrate, and silica are also present in the formulation to promote stain removal [13]. Whitening toothpastes can also contain enzymes that chemically modify the extrinsic stain, which may reduce the intensity and appearance of staining [9]. Optical modifying toothpastes containing colorants, such as proprietary Blue Covarine, a well-known phthalocyanine dye [14], are claimed to work by depositing a thin, semitransparent film of bluish pigment on the dental surface [15]. Colorants are speciﬁc dyes intended to give teeth a whiter color [11]. Incident light interacts with the tooth structure with a resulting whiter and brighter appearance due to the tooth color shift from yellow to blue (i.e., reduction in *b*^*∗*^), giving an overall self-perception of tooth whiteness [15]. However, more research is still needed to evaluate the efficacy of whitening toothpastes containing colorants on the optical properties of enamel as their interactions with the tooth structure and their effects/alterations on stained teeth are still unclear.

This in vitro study aimed to investigate the whitening potential of commercially available and experimental toothpastes containing optical colorants in their formulations. The present study also evaluated the chemical and physical characteristics of these toothpastes, and their consequences on the enamel surface, the morphological changes on the enamel surface, and the elemental analysis of the enamel after treatment. The study's hypothesis was that toothpastes containing colorants would produce less chromatic change than commercial whitening toothpastes.

## 2. Materials and Methods

### 2.1. Experimental Design

In this in vitro study, the chromatic change of the enamel surface of bovine teeth after treatment with different bleaching toothpastes was evaluated. The sample included 125 bovine incisors. The specimens were randomly divided into five experimental groups, as described in Table 1 (*n* = 25). Table 1 also depicts the composition of the toothpastes selected for the study.

Other outcome variables were the chemical and physical characteristics of the toothpastes and the morphological changes on enamel surface, as well as the elemental analysis of the enamel (*n* = 3) after treatment. Toothpastes were selected among commercial products indicated as bleaching toothpastes by the manufacturers. Two of the toothpastes are experimental products containing a photosensitizer phthalocyanine and blue silica-PHT and SDG toothpastes, respectively.

### 2.2. Specimen Preparation for Bleaching Analysis

A total of 125 recently extracted sound bovine teeth were selected and cleaned with a slurry of pumice/water and then stored in a 0.1% thymol solution at room temperature. Software (G^*∗*^ Power NT, USA) was used to determine the minimum sample size according to these parameters: 95% statistical significance, 0.80 test power, 0.40 effect size, and 5 experimental groups. This resulted in a minimum sample size of 125 specimens (*n* = 25/group). Selection criteria for this study were similar shade, size, and surface texture. The specimens also presented an initial Vita A4 shade, determined using a previously calibrated portable reflection spectrophotometer (SpectroShade Micro-MHT Medical High Technology, Italy), employed against a white background. The surface enamel was free of alterations, such as cracks, hypoplasia, or hypomineralization.

### 2.3. Enamel Treatment with Bleaching Toothpastes

Toothpaste slurries (1 g; 1 : 3, w/v in distilled water; ISO 11609-2017) were applied to the cervical third area of the labial surface of bovine teeth using a calibrated syringe. Then, the specimens were brushed using an electric toothbrush for 5 minutes (120 cycles per minute, 22°C) [16], three times a day for 14 days. The toothbrushes were fixed in a device that allowed the heads to be aligned parallel to the specimens' surfaces [17]. The specimens were brushed in linear motion with an axial load of 200 g. After each brushing cycle, the specimens were washed in running water for 5 seconds.

### 2.4. Color Change Analysis

The collection of data was performed according to the CIE-Lab (Commission Internationale de l'Eclairage *L*^*∗*^, *a*^*∗*^, *b*^*∗*^) color space with a portable reflection spectrophotometer (SpectroShade Micro-MHT Medical High Technology, Italy) employed against a white background. The device was previously calibrated using white and green standards according to the manufacturer's instructions. The coordinate system translates the CIE luminosity (*L*^*∗*^) numerically and matches it in red (+*a*^*∗*^) or green (−*a*^*∗*^) and yellow (+*b*^*∗*^) or blue (−*b*^*∗*^). Images of the cervical third area of the labial surface of the specimens were obtained. Then, using the appropriate software, the cervical third area was delimitated and the values of *L*^*∗*^, *a*^*∗*^, and *b*^*∗*^ were obtained. The color change was evaluated according to the color system CIE-*L*^*∗*^*a*^*∗*^*b*^*∗*^, in which “*L*” indicates color luminosity (0-black to 100-white); *a*^*∗*^, the amount of red (positive values) and green (negative values); *b*^*∗*^, the amount of yellow (positive values) and blue (negative values). Prior to the treatment, initial color readings in the selected area were obtained (baseline data) and then repeated after brushing cycles with the bleaching toothpastes for 14 days. Three color measurements were taken on each specimen. The specimens were kept at room temperature in distilled water between the brushing cycles. For collecting the data, the specimens were previously blot-dried with absorbent paper. The differences (final value minus initial) between the CIE-Lab coordinates (Δ*L*^*∗*^, Δ*a*^*∗*^, and Δ*b*^*∗*^) were calculated for each experimental group. Then, the whiteness index for dentistry (*WI*_*D*_), whiteness index differences (Δ*WI*_*D*_), color change (Δ*E*_*ab*_), and CIEDE2000 color difference (Δ*E*_00_) were determined according to the following equations [19–21]:

(1) $$\begin{array}{c} WI_{D}=0.511L^{*}-2.324a^{*}-1.100b^{*}, \end{array}$$

(2) $$\begin{array}{c} \Delta WI_{D}=WI_{D}\text{sample}-WI_{D}\text{baseline}, \end{array}$$

(3) $$\begin{array}{c} \Delta E_{ab}^{*}={\left[{\left(\Delta L^{*}\right)}^{2}+{\left(\Delta a^{*}\right)}^{2}+{\left(\Delta b^{*}\right)}^{2}\right]}^{1/2}, \end{array}$$

(4) $$\begin{array}{c} \Delta E_{00}={\left[{\left(\frac{\Delta L\prime }{K_{L}S_{L}}\right)}^{2}+{\left(\frac{\Delta C\prime }{K_{C}S_{C}}\right)}^{2}+{\left(\frac{\Delta H\prime }{K_{H}S_{H}}\right)}^{2}+RT^{*}{\left(\frac{\Delta C\prime }{K_{C}S_{C}}\right)}^{*}\left(\frac{\Delta H\prime }{K_{H}S_{H}}\right)\right]}^{1/2}, \end{array}$$

where ∆*L*^*∗*^, ∆*a*^*∗*^, and ∆*b*^*∗*^ are the difference in the respective values before and after treatment, Δ*L*, Δ*C* e ΔH represent differences between initial and final lightning, chroma, and hue values. *RT* explains the interaction between chroma and hue differences in the blue region. *KL*, *KC*, and *KH* are correction terms for experimental conditions set to a value of 1 [18]. A single trained evaluator performed all measurements. The tooth whitening efficacy was interpreted as a function of the whitening-dependent color differences as follows [22]: (1) not effective; (2) moderately effective; (3) good effectiveness; (4) very good effectiveness; (5) excellent effectiveness.

Characterization of the enamel superficial morphology and cross-sections was performed by scanning electron microscopy (SEM) imaging observation and energy-dispersive X-ray spectroscopy (EDS).

Three additional enamel specimens were prepared for morphology analysis of the surface treated with different toothpaste slurries using a scanning electron microscope (10 kV, SEM, JSM-5310; JEOL, Tokyo, Japan). The specimens were dehydrated in silica gel for 3 h. The specimens were previously sputter-coated with gold in a vacuum evaporator (MED 010; Balzers, Balzers, Liechtenstein). Then, the specimens were microscopically analyzed, obtaining photomicrographs. Representative images of selected regions were obtained (×5000 magnification). The EDS point analysis (80 mm^2^, SDD, Oxford Instruments, Concord, MA, USA) determined the qualitative elemental analysis operating in high vacuum mode and an accelerating voltage of 15 kV. The mean values obtained at three points per sample (300 *µ*m^2^ per point) were calculated.

Enamel cross-sections were obtained for the analysis of the subsurface after longitudinally sectioning the specimens under water cooling. The enamel halves were dehydrated in silica gel for 3 h and then the elemental analyses were performed. The specimens were then gold-sputtered for the SEM analysis.

### 2.5. Physicochemical Analyses of the Toothpaste Slurries

Toothpaste slurries were prepared, and the pH was immediately determined in three different samples using a pH electrode (AK95, Akso, São Leopoldo, RS, Brazil), calibrated with standards (pH 4.0 and 7.0). The slurries were then diluted in distilled water (250 mg/L). The particle size distribution, effective diameter, zeta potential, and polydispersity index were also evaluated at room temperature (Phase Analysis Light Scattering–PALS, 90 Plus, Brookhaven Instruments Corporation, Holtsville, NY, USA) [16, 23, 24]. Ten replications were evaluated (*n* = 10).

### 2.6. Statistical Analysis

Means and standard deviations of the whiteness index difference (Δ*WI*_*D*_), color change difference (Δ*E*_*ab*_), and CIEDE2000 color difference (Δ*E*_00_) were calculated and statistically analyzed (Statsoft, Tulsa, OK, USA). The normal distribution of the variables was previously evaluated with the Kolmogorov–Smirnov and Levene tests. The data were subjected to one-way analysis of variance, followed by Tukey test (*α* = 0.05).

## 3. Results

In Table 2, the means and the standard deviation of the whiteness index difference (Δ*WI*_*D*_), color change difference (Δ*E*_*ab*_), and CIEDE2000 color difference (Δ*E*_00_) results are displayed. SDG presented a higher whitening effect (Δ*WI*_*D*_) and SWS presented a lower mean. SDG and PHT presented significantly higher means when compared to STW and SWS (*p* < 0.05). No significance was observed when the means of WRP and STW were compared (*p* > 0.05). In general, regarding the analyses of the color change (Δ*E*_*ab*_ and Δ*E*_00_), the statistical analysis demonstrated that the groups treated with experimental whitening toothpastes (SDG and PHT) exhibited higher chromatic change, while STW and SWS, lower. In contrast with the other experimental groups (Table 2), WRP exhibited intermediary chromatic change. In this manner, in both color change analyses, the tooth whitening efficacy of the experimental whitening toothpastes SDG and PHT was graded with a 4, corresponding to very good effectiveness. On the other hand, the toothpastes WRP, STW, and SWS were graded with a 3, which corresponds to good effectiveness.

**Figure 1:**
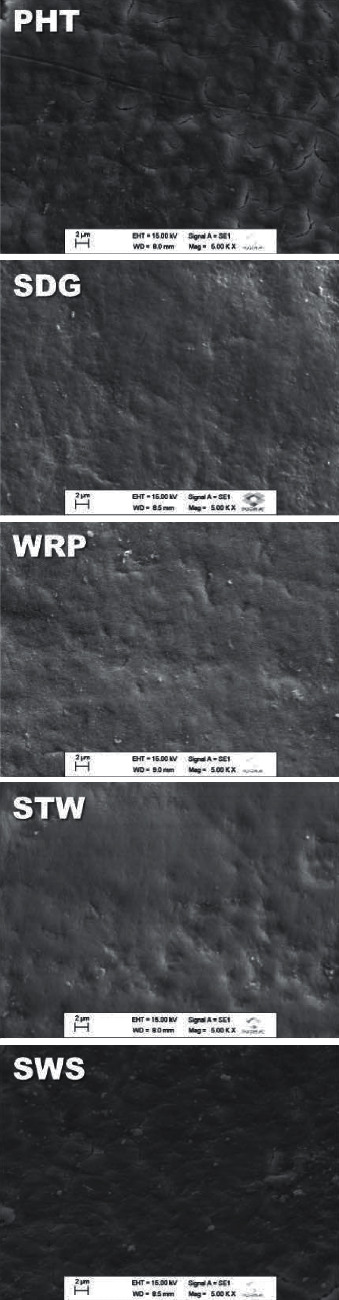
Representative images of Scanning Electron Microscopy analysis in different groups.

Figure 1 shows the representative scanning electron micrographs of the enamel surface, and the respective elemental mapping is displayed in Table 3. In general, surface irregularities in all groups treated with different toothpastes were observed in the morphological analysis of the enamel. Despite the presence of Si in all products, EDS analysis detected stronger silicon signals in the experimental product SDG (Table 3).

**Figure 2:**
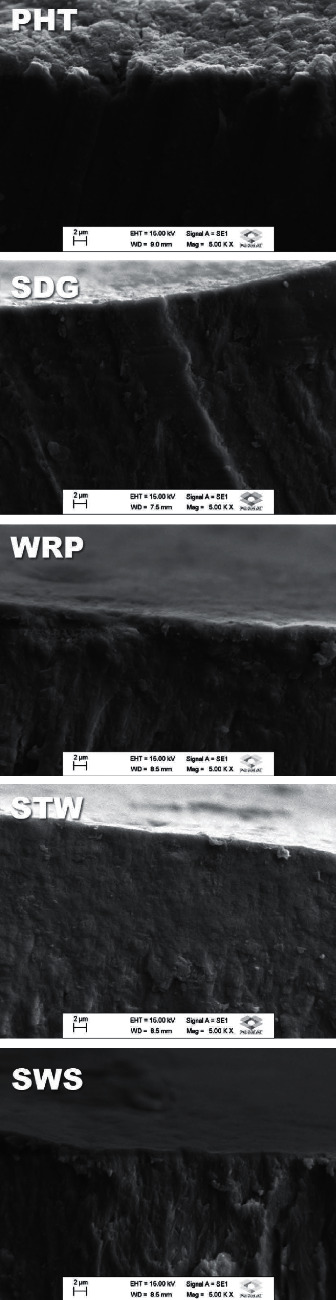
Representative scanning electron micrographs of the enamel cross-sections as a function of the experimental group.

Figure 2 shows a cross-sectional area of the enamel treated with the different toothpastes; for comparison, SDG shows a representative scanning electron micrograph of the remineralized cross-sectional area with a silicon-rich mineral layer. The amounts of sodium, phosphorus, and calcium also varied as a function of the treatments with different products. Variability in the Ca/P ratio observed in the analyzed specimens can also be seen in Table 4, with the lowest ratio for SWS (1.58) and the highest for SDG and WRP (2.04).

The results of the chemical and physical properties of the toothpaste slurries are displayed in Table 4. Two of the toothpastes presented alkaline pH (WRP and STW), whereas the other two products had acid pH (SDG and SWS). Only the toothpaste PHT presented a neutral pH. The particle size varied from 266.2 nm (STW) to 412.8 nm (PHT). The zeta potential varied from −0.84 mV (SWS) to 1.37 mV (STW), whereas the polydispersity index varied from 0.18 (STW and SWS) to 0.34 (SDG).

## 4. Discussion

The results of the present study demonstrated that the experimental groups treated with the photosensitizer-containing toothpaste (PHT) and the experimental blue silica-containing REFIX technology toothpaste (SDG) exhibited significantly higher means of Δ*WI*_*D*_. Similar results were observed when treated with WRP, but no significance was observed when compared to the group treated with STW (*p* > 0.05). It has been reported that higher values of the whiteness index difference for dentistry (Δ*WI*_*D*_) are an indication of whiter teeth, whereas lower means represent darker teeth [25]. In the present study, all experimental groups presented positive Δ*WI*_*D*_ results, which indicates the whitening ability of the whitening toothpastes [21]. Although Δ*WI*_*D*_ is an important parameter that correlates color with proximity to white, the interpretation of the results needs to be associated with other variables, such as Δ*E*_*ab*_ and Δ*E*_00_, which consider the color changes based on the *L*^*∗*^, −*a*^*∗*^, −*b*^*∗*^ parameters [21].

The results of the present study also confirmed that SDG and PHT exhibited significantly higher means of Δ*E*_*ab*_ and Δ*E*_00_ when compared to other groups. The only exception was for WRP, which exhibited similar results of Δ*E*_ab_ when compared to SDG and PHT. Based on the results of Δ*E*_*ab*_ and Δ*E*_00_, SDG and PHT were graded with a 4 in terms of tooth whitening efficacy, which means that these whitening toothpastes present very good effectiveness. The other toothpastes (WRP, STW, and SWS) were graded with a 3, which corresponds to good effectiveness. In this manner, the first hypothesis, which anticipated that toothpastes containing photosensitizer components would produce lower chromatic changes relative to the commercial whitening toothpastes, was not accepted. In spite of these differences, the means of Δ*E*_*ab*_ were higher than 2.7 in all experimental groups, which is the previously established limit for clinically observing color changes [26].

The rationale of the use of photosensitizers in whitening toothpastes relies on the fact that active oxygen species are produced through these photochemical reactions, which seems to transfer energy to the oxygen molecule in an excited singlet state (singlet oxygen) that has a high chemical reactivity [27, 28]. In this PDT-based whitening toothpastes, a photosensitive dye is excited by light [29]. In this toothpaste was added a self-activated iron phthalocyanine [30]. In the excited state, these dyes can donate highly energetic electrons and reduce O_2_ and H_2_O_2_ [31]. Photodynamic whitening enables the formation of hydroxyl radical and singlet oxygen, regarded as potent bleaching whitening agents formed in situ, being able to decompose organic materials [31].

Recently, attempts have been also made to enhance the deposition of brighter pigment on the tooth surface for immediate whitening benefits in oral care products. It has been demonstrated that the bluish shift of the yellow-blue axis (*b*^*∗*^) is more relevant for the perception of whiter and brighter teeth than an increase in luminosity (*L*^*∗*^) or changes in the green-red axis (*a*^*∗*^) within the CIE-Lab color space [32]. In this manner, using the blue-colored phthalocyanine, similar to that found in the toothpaste containing proprietary Blue Covarine technology, would provide the visual perception of whiter teeth enhanced by the deposition of the dye. Based on the manufacturer's information, this silica-based whitening toothpaste was formulated to contain an abrasive system to provide an effective extrinsic staining removal. Recently published papers in which the effectiveness of pigments of optical effect in tooth whitening was evaluated demonstrated controversial results, with either improved whitening results [15, 33] or no whitening effects [6, 9].

The toothpaste PHT contains the proprietary PHTALOX technology, a derivative of the iron phthalocyanine [30], which is similar to that found in the Blue Covarine-containing toothpastes (copper phthalocyanine) [14]. Considering that these ions were found on the surface of the brushed enamel, it could be speculated that there might be a deposition of lighter pigments on the enamel surface, considering that this ion was detected in the EDS analysis, which is the central metal atom in the phthalocyanine molecular structure [34]. Conversely, SDG contains the proprietary REFIX technology with experimental blue silica. REFIX technology-containing toothpaste was regarded to allow the formation of a silicon-enriched hydroxyapatite layer on the enamel surface [35, 36]. Silica is a component of bioactive glass and acts as a nucleation site for calcium and phosphate ion precipitation in the formation of hydroxyapatite [37]. Although the presence of silicon in all whitening toothpastes was demonstrated, a higher % was found in the experimental toothpaste SDG (0.72) (Table 3), with REFIX technology. Silica particles promote more rapid release of Ca and P [38]. Replacing phosphate ions (PO_4_) in the molecular structure of hydroxyapatite with silicate ions (SiO_4_) leads to the formation of calcium silicate, which is deposited onto the enamel surface [39]. It can be speculated that the silicon-rich layer formed on the surface enamel seems to induce an optical color change with a whitening effect. In the toothpaste SDG, the only source of silicon is the blue silica, which allowed the formation of this surface rich of silicon, which favored the change in the optical properties of the enamel.

The commercial whitening toothpastes tested in the present study contain no peroxide as an ingredient. In this manner, these whitening products seem to have a focus on the staining removal during brushing with toothpastes considering the presence of solid particles such as titanium dioxide (WRP and STW) and silicates, such as mica and bentonite (SWS). Toothpastes WRP and STW also contain active ingredients against dentin hypersensitivity, which represents a specific choice in oral care for patients wishing brighter teeth. The abrasiveness of toothpaste varies with the particle hardness, size, shape, and pH [40]. As previously described [16], depending on the zeta potential of the particles, there may be a propensity of particle aggregation in an electrically neutral zeta potential, leading to the formation of larger-sized particles, with a consequent tendency for particle sedimentation [41]. This leads to an improved staining removal and ultimately to increased enamel roughness and/or dentin hypersensitivity, depending on the agglomerated particle size [16]. In the present study, the zeta potential means varied as functions of the toothpastes, with none of them displaying a tendency to aggregation (Table 4). The experimental whitening toothpastes PHT and SDG presented bigger particle sizes and lower stability, considering that the ideal polydispersity index would lower than 0.20 [42]. In this manner, it can be speculated that the significantly higher color changes obtained after treatment with PHT and SDG can be due to the synergism in the presence of a photosensitizer and blue silica in the composition, associated with the physical and chemical parameters that simultaneously contributed to change the optical characteristics of the treated enamel.

The differences in the outcomes observed in the present study support the speculation that chromatic changes of the enamel may occur due to the modification of the hydroxyapatite structure induced by the treatment with whitening toothpastes. As previously demonstrated [35], depending on the substitution at the sites of the hydroxyapatite structure, changes in calcium phosphate nucleation, hydroxyapatite growth and crystallization, and its stability may occur in an oral environment rich in ions released by the toothpaste [43]. In this manner, changes in the mineral phase of teeth may occur in terms of stoichiometric hydroxyapatite. A calcium deﬁcient hydroxyapatite is claimed to have a nonstoichiometric apatite (Ca/P < 1.67), with small crystals and poor crystallinity, leading to a relatively high solubility [44]. In the present study, all whitening toothpastes presented Ca/P ratios higher than 1.74 (SWS) (Table 4). The experimental toothpastes PHT and SDG exhibited a Ca/P ratio of 1.86 and 2.04, respectively.

In general, commercial whitening toothpastes improve tooth whiteness by the gradual removal and/or control of extrinsic stains over time [5]. It is expected that these over-the-counter products would beneﬁt patients with gradual, long-term dental whitening [31]. It is true that most stains on the enamel surface can be easily removed by professional cleaning. However, patients tend to obtain more from whitening treatments depending on their clinical case. In these cases, whitening toothpaste can be an ideal option recommended by clinicians. The reasons that explain the addition of different compounds to the formulation of toothpastes of this category rely on the fact that the whitening mechanism using brushing associated with toothpastes comprises a low cost, less aggressive, slower-pace whitening treatment, compared to the treatment with gels containing hydrogen peroxide in-office and/or at-home whitening systems [13]. Concerns have been raised about the biological risks of tooth whitening with peroxides [45] and whitening toothpastes have been considered as relevant alternative clinical options for clinicians and patients [46]. Conversely, in order to simplify the technique and increase the whitening effect, an easier and safer alternative was possible with the use of whitening toothpastes, developed with peroxide-free formulations. Whitening toothpastes are regarded as removing and/or controlling extrinsic stains with optimized abrasives, surfactants, polyphosphates, and enzymes [1]. In this manner, compounds such as colorants were added to the formulation to boost the whitening treatment with whitening toothpaste.

The present study investigated the whitening potential of experimental toothpastes containing optical colorants in their formulations in comparison with commercial products. The chemical and physical characteristics of the toothpastes, the morphology, and elemental analysis of the enamel surface after treatment were also analyzed. Considering its limitations, the present study could be performed on human teeth instead of bovine teeth. In addition, it could also be argued that in this *in vitro* study, no direct light exposure was performed on the specimens to activate the photosensitizer and consequently lead to photoreactions, which would produce active species. Another limitation could be testing the toothpastes after coffee staining of the specimens, which could provide important information in terms of their effectiveness after this color challenge. Conversely, it has been claimed that daylight can also be used as a light source in PDT [47]. In addition, the PHTALOX-containing photosensitizer toothpaste is self-activated. Although daylight cannot penetrate deeper into the dental tissues and its emission spectrum contains a large proportion of blue light, this therapy using either photosensitizer or silica blue can be effective for superﬁcial treatments. Even under *in vitro* conditions, the ambient light may provide the full spectrum of natural light. In this manner, it can be speculated that better results would be obtained under clinical conditions. Further studies are needed to investigate the performance of toothpaste containing optical colorants in *in situ* and *in vivo* studies on the effectiveness of whitening toothpastes on dental substrates.

## 5. Conclusions

Based on the findings of this study, it can be stated that whitening toothpastes containing optical colorants are efficient for whitening the enamel, considering that whiter teeth were observed following treatment (higher means of Δ*WI*_*D*_). In addition, these toothpastes were considered to present very good effectiveness (grade 4) when the parameters Δ*E*_*ab*_ and Δ*E*_00_ were evaluated. The tooth whitening effect after brushing with whitening toothpastes containing optical colorants seems to be favorably affected by the synergism among the chemical and physical characteristics studied.

## Figures and Tables

**Table 1:** Composition of the toothpastes selected for the study^*∗*^.

Product	Ingredients	Active agents	Lot^#^ Exp. date
Experimental PHTALOX dental gel^a^ (PHT)	Glycerin, silica, sorbitol, sodium lauryl sulfate, aqua, aroma, PEG-12, cellulose grum, xylitol, sodium sacharin, triclosan, menthol, mica, sodium benzoate.	1450 ppm F- (as sodium fluoride)	N/A
		Tetrasodium pyrophosphate	
		Iron phthalocyanine chloride (PHTALOX)	

Experimental blue silica dental gel^b^ (SDG)	Glycerin, silica, sorbitol, sodium lauryl sulfate, aqua, aroma, PEG-12, cellulose grum, O-Phosphoric acid, xylitol, sodium sacharin, triclosan, menthol, mica, sodium benzoate, nanoparticulate sorbosil BFG 51 (blue silica).	1450 ppm F- (as sodium fluoride)	N/A
		Tetrasodium pyrophosphate	
		REFIX® technology	

Sensodyne whitening repair & protect^c^ (WRP)	Glycerin, PEG-8, hydrated silica, calcium sodium phosphosilicate (NOVAMIN), cocamidopropyl betaine, sodium methyl cocoyl taurate, aroma, titanium dioxide, carbomer, silica, sodium saccharin, limonene.	1450 ppm F- (as sodium fluoride)	T60010658502 05/2020
		NovaMin® technology	

Sensodyne true white^d^ (STW)	Sorbitol, aqua, glycerin, hydrated silica, pentasodium triphosphate, potassium nitrate, peg-6, cocamidopropyl betaine, aroma, titanium dioxide, xanthan gum, sodium hydroxide, sodium saccharin, limonene.	1450 ppm F- (as sodium fluoride)	XL0204V 09/2020
		Potassium nitrate 5% w/w	

Snow white toothpaste^e^ (SWS)	Aqua, hydrated silica, glycerin, sorbitol, titanium dioxide, bentonite, hydroxyapatite, decyl glucoside, mica, aroma, cocamidopropyl betaine, tocopherol, xanthan gum, potassium chloride, sodium saccharin, curcuma xanthorrhiza root extract, aloe barbadensis juice extract, sodium hydroxide, glucose oxidase, sodium benzoate, tin oxide, maltodextrin, silica, d-limonene, linalool, eugenol.	950 ppm F- (as sodium monofluorophosphate	P2/L1 N/A

^*∗*^Manufacturers' information. ^a,b^ Rabbit corp, Londrina, PR, Brazil; ^c,d^ Glaxosmithkline Brazil Co., ^e^ Curaden AG, Kriens, Lucerne, Switzerland; ^f^ Unilever UK Limited, Leatherhead, Surrey, UK.

**Table 2:** Means and standard deviation (SD) of the whiteness index difference (Δ*WI*_*D*_), color change difference (Δ*E*_*ab*_), and CIEDE2000 color difference (Δ*E*_00_) results as functions of Δ*L*, Δ*a*, and Δ*b*.

	Δ*L*	Δ*a*	Δ*b*	Δ*WI*_*D*_	Δ*E*_*ab*_	Δ*E*_00_	Rating and interpretation Δ*E*_*ab*_^*∗*^	Rating and interpretation Δ*E*_00_^*∗*^
PHT	3.7 (2.0)	−1.2 (0.6)	3.4 (2.8)	8.4^A^ (4.2)	5.7^A^ (2.6)	3.8^A,C^ (1.6)	4	4

SDG	4.6 (1.5)	−1.3 (0.9)	3.4 (2.9)	9.0^A^ (6.0)	6.5^A^ (1.7)	4.3^A^ (1.2)	4	4

WRP	2.7 (1.2)	−1.0 (0.7)	3.7 (2.6)	7.7^A,C^ (4.1)	5.0^A,C^ (2.4)	3.0^B,C^ (1.2)	3	3

STW	2.5 (1.9)	−0.4 (0.4)	2.2 (2.4)	4.5^B,D^ (4.3)	4.1^B,C^ (2.0)	2.6^B,C,E^ (1.3)	3	3

SWS	1.9 (1.1)	−0.2 (0.6)	1.6 (1.5)	2.9^B^ (3.5)	2.9^B,D^ (1.3)	1.9^B,D,E^ (0.9)	3	3

*N* = 25 evaluated teeth per experimental group. Capital letters compare enamel color changes (Δ*WI*_*D*_, Δ*E*_*ab*_, and Δ*E*_00_) at each time point (columns), according to one-way ANOVA and Tukey post hoc test (Δ*WI*_*D*_, Δ*E*_*ab*_, and Δ*E*_00_). Different capital letters, A and B; C and D; E and F: significant (*p* < 0.05). ^*∗*^Grades: (1) not effective; (2) moderately effective; (3) good effectiveness; (4) very good effectiveness; (5) excellent effectiveness [22].

**Table 3:** Elemental mapping by weight% according to the experimental groups.

Element	PHT	SDG	WRP	STW	SWS
C	12.38	9.20	4.95	9.06	17.22
O	29.42	29.03	18.97	30.52	35.18
Na	0.45	0.43	0.29	0.41	0.27
Mg	0.83	0.56	0.27	0.35	0.82
Si	0.25	0.72	0.32	0.19	0.27
P	12.59	11.63	16.74	12.77	10.31
K	0.00	0.24	0.00	0.12	0.00
Cl	0.41	0.00	0.39	0.15	0.00
Ca	23.46	23.78	34.11	24.53	17.94
Sr	0.00	0.00	1.57	1.10	0.83
Sn	0.00	0.00	0.00	0.00	0.44
Fe	0.30	0.00	0.00	0.00	0.00
Ca/P	1.86	2.04	2.04	1.92	1.74

**Table 4:** Results of the chemical and physical analyses of the toothpaste slurries.

Product	pH^*∗*^	Particle size (nm)	Zeta (mV)	Polydispersity index
PHT	7.12	412.8 (9.6)	0.71 (0.33)	0.31 (0.03)
SDG	4.86	386.2 (14.1)	−0.12 (0.37)	0.34 (0.01)
WRP	9.70	353.5 (10.3)	0.48 (0.47)	0.19 (0.03)
STW	8.76	266.2 (13.9)	1.37 (1.07)	0.18 (0.03)
SWS	5.85	297.3 (9.5)	−0.84 (0.50)	0.18 (0.03)

^*∗*^pH of the slurry.

## Data Availability

The data used to support the findings of this study are included within the article.
